# P-206. Reducing Hospital Acquired *Clostridioides difficile* Infections: A Comparison of Two Equally Effective Testing Strategies

**DOI:** 10.1093/ofid/ofae631.410

**Published:** 2025-01-29

**Authors:** Elizabeth Nothdurft, Nirmol Philip, J William Campbell, Elysa Sirtak, Madison Martin-Giacalone, Mackenzie Martin, Emily Leinauer, Brenden Giblin

**Affiliations:** St. Luke's Hospital, Chesterfield, Missouri; St. Luke's Hospital, Chesterfield, Missouri; St. Luke's Hospital, Chesterfield, Missouri; St. Luke's Hospital, Chesterfield, Missouri; St. Luke's Hospital, Chesterfield, Missouri; St. Luke's Hospital, Chesterfield, Missouri; St. Luke's Hospital, Chesterfield, Missouri; St. Lukes Hospital, Chesterfield, Missouri

## Abstract

**Background:**

A positive *Clostridioides difficile* (*C. diff*) test obtained after day three of admission qualifies as hospital acquired *C. diff* infection (HA-CDI). Given PCR’s high sensitivity for *C. diff*, the decision to test necessitates clinical judgement to avoid false positive results that may lead to increased nosocomial infection rates, unnecessary treatment, and increased financial burden. This study investigated two interventions to decrease unnecessary *C. diff* testing after day three of admission.

**Figure:**
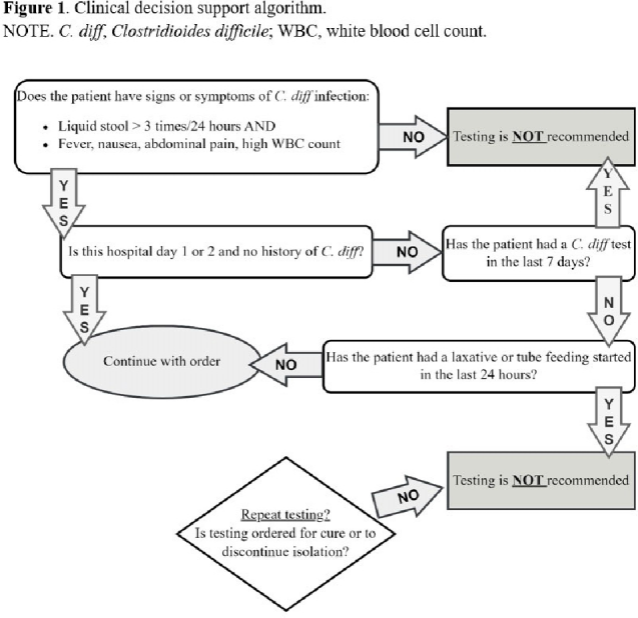

**Methods:**

A multistep testing algorithm, which consists of an initial enzyme immunoassay for glutamate dehydrogenase (GDH) and Toxin A/B testing followed by *C. diff* PCR test by a reflex, is utilized in our institution. To ensure appropriate *C. diff* test ordering, our Infection Control (IC) team implemented two stages of interventions during a span of five years. In June 2019, Stage 1 involved a hard stop and manual review of all *C. diff* test orders placed after three midnights from Monday through Friday, requiring an IC or infectious diseases team member to approve these *C. diff* testing requests. Orders were cancelled if all established testing criteria were not met (Figure 1). In January 2023, Stage 2 was implemented which replaced manual review with an algorithm in electronic medical record (EMR).

Outcomes were measured by examining National Healthcare Safety Network (NHSN) LabID data and the standardized infection ratio (SIR) before and after each stage implementation.

**Figure:**
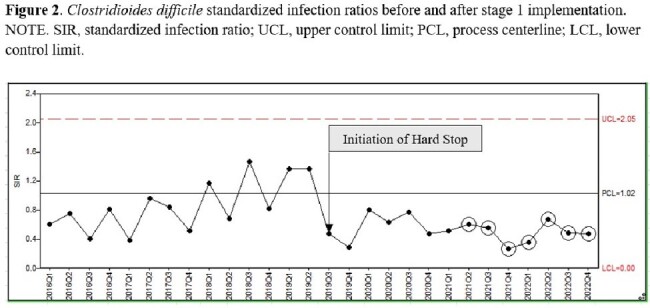

Stage 1 SIR

**Results:**

From 2019 to 2023, our testing interventions resulted in a reduction in HA-CDI incidence. Stage 1 implementation significantly decreased average HA-CDI incidence from 9.3 to 4.06 infections/1000 patient days (PD) (p< 0.0001) resulting in a decreased average HA-CDI SIR from 1.25 to 0.52 (p< 0.0001).

Further HA-CDI decrease resulted after Stage 2 implementation with HA-CDI rate decreasing from 4.06 to 3.29 infections/1000 PD (p=0.656) and with HA-CDI SIR decreasing from 0.52 to 0.48 (P =0.172) (Figure 2, 3). This decline was not statistically significant.

**Figure:**
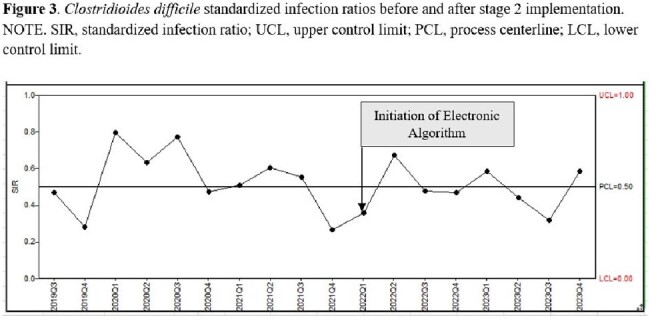

Stage 2 SIR

**Conclusion:**

Manual testing review and EMR-based HA-CDI algorithm implementation effectively decreased the number of positive HA-CDI test results. Use of the electronic algorithm greatly reduced the workload burden on our IC team with no increase in HA-CDI.

**Disclosures:**

**Elizabeth Nothdurft, PharmD, BCPS, BCIDP**, Abbvie: Honoraria **Nirmol Philip, MD, MPH**, Nestle: Honoraria

